# Using Facial Micro-Expressions in Combination With EEG and Physiological Signals for Emotion Recognition

**DOI:** 10.3389/fpsyg.2022.864047

**Published:** 2022-06-28

**Authors:** Nastaran Saffaryazdi, Syed Talal Wasim, Kuldeep Dileep, Alireza Farrokhi Nia, Suranga Nanayakkara, Elizabeth Broadbent, Mark Billinghurst

**Affiliations:** ^1^Empathic Computing Laboratory, Auckland Bioengineering Institute, The University of Auckland, Auckland, New Zealand; ^2^Augmented Human Laboratory, Auckland Bioengineering Institute, The University of Auckland, Auckland, New Zealand; ^3^Department of Psychological Medicine, The University of Auckland, Auckland, New Zealand

**Keywords:** emotion recognition, electroencephalography (EEG), facial micro-expressions, physiological signals, neural networks, decision fusion, OpenBCI

## Abstract

Emotions are multimodal processes that play a crucial role in our everyday lives. Recognizing emotions is becoming more critical in a wide range of application domains such as healthcare, education, human-computer interaction, Virtual Reality, intelligent agents, entertainment, and more. Facial macro-expressions or intense facial expressions are the most common modalities in recognizing emotional states. However, since facial expressions can be voluntarily controlled, they may not accurately represent emotional states. Earlier studies have shown that facial micro-expressions are more reliable than facial macro-expressions for revealing emotions. They are subtle, involuntary movements responding to external stimuli that cannot be controlled. This paper proposes using facial micro-expressions combined with brain and physiological signals to more reliably detect underlying emotions. We describe our models for measuring arousal and valence levels from a combination of facial micro-expressions, Electroencephalography (EEG) signals, galvanic skin responses (GSR), and Photoplethysmography (PPG) signals. We then evaluate our model using the DEAP dataset and our own dataset based on a subject-independent approach. Lastly, we discuss our results, the limitations of our work, and how these limitations could be overcome. We also discuss future directions for using facial micro-expressions and physiological signals in emotion recognition.

## 1. Introduction

Human emotions involve numerous external and internal activities and play an essential role in our daily life. Facial expressions, speech, and body gestures are some of the external activities affected by emotional situations. Changes in brain activity, heart rate, blood pressure, respiration rate, body temperature, and skin conductance are examples of internal emotional effects (Verma and Tiwary, 2014). Nowadays, we are surrounded by digital characters, intelligent devices, and computers in the modern world. There is a need for better interaction with these systems, and it is becoming increasingly important to recognize emotions in many human-human and human-computer interactions (Zheng et al., 2018). The effectiveness of our remote interactions, therapy, consultations, or training sessions could be improved if they were equipped with emotion recognition systems. For example, recognizing emotion in remote e-learning (Khalfallah and Slama, 2015) could enhance the performance of learning. Similarly, in Empathic Computing applications, the goal is to measure the emotions of people teleconferencing together and use the result to improve remote communications (Piumsomboon et al., 2017).

Finally, creating intelligent agents with emotion recognition capabilities could be helpful in health care, education, entertainment, crime investigation, and other domains (Huang et al., 2016). It could be beneficial for intelligent assistants (Marcos-Pablos et al., 2016) or humanoid robots (Bartlett et al., 2003) to be able to measure the emotions of their users. Zepf et al. (2020) discuss the importance of emotion-aware systems in cars. Similarly, Hu et al. (2021) presented a conversational agent that recognizes emotions based on the acoustic features of speech. According to Chin et al. (2020) empathy between conversational agents and people can improve aggressive behavior. Schachner et al. (2020) discussed developing intelligent conversational agents for health care, especially for chronic diseases. Similarly, Aranha et al. (2019) reviewed software with smart user interfaces capable of recognizing emotions in various fields, including health, education, security, and art. According to their review, emotion recognition has often been used for adjusting sounds, user interfaces, graphics, and content based on user emotion.

Facial expressions are one of the most commonly used input modalities analyzed to identify emotional state (Sun et al., 2020). They are used in many HCI applications (Samadiani et al., 2019). Although studies have shown significant results in recognizing emotion from facial expressions (Li and Deng, 2020), using these methods in daily life faces some challenges because they can be controlled or faked by humans (Hossain and Gedeon, 2019). Many methods for recognizing emotions from facial expressions are based on datasets with non-spontaneous facial expressions or exaggerated facial expressions which do not correctly reflect genuine emotions (Weber et al., 2018; Li and Deng, 2020). In the real world, people usually show subtle involuntary expressions (Zeng et al., 2008) or expressions with lower intensity according to the type of stimuli. These studies show the importance of developing and improving robust methods for recognizing spontaneous emotions.

### 1.1. Recognizing Spontaneous Emotions

Three main approaches have been proposed in the literature for recognizing subtle, spontaneous emotions in the real world, which are listed as follows:

- Extracting involuntary expressions from faces.
- Using physiological signals that cannot be faked.
- Using a combination of various input modalities.

#### 1.1.1. Extracting Facial Micro-Expressions From Faces

**Figure 1:**
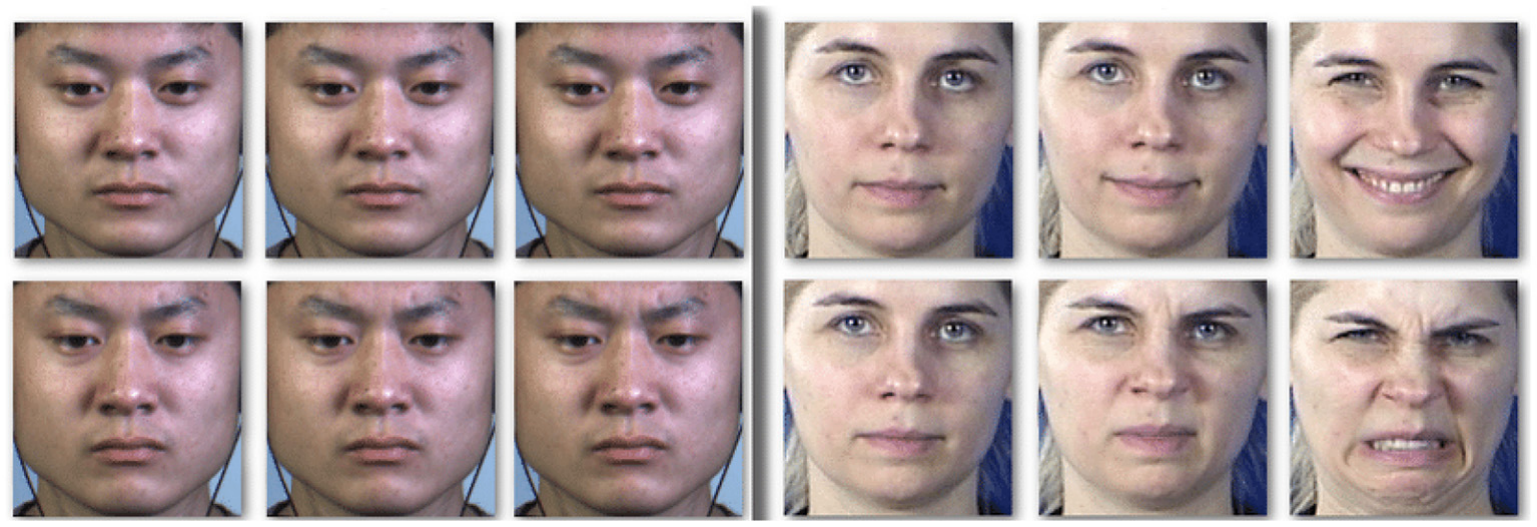
Facial micro-expressions compared to facial macro-expressions. Facial micro-expressions **(left)** and macro expressions **(right)** for happiness (line1) and disgust (line2), from CASME II (Pantic et al., 2005) and MMI (Yan et al., 2014) datasets (Allaert et al., 2018).

In this approach, the focus is on extracting facial micro-expressions instead of facial macro-expressions. Facial macro-expressions or intense facial expressions are voluntary muscle movements in the face that are distinguishable, cover a large area of the face, and their duration is between 0.5 and 4 s (Ekman and Rosenberg, 1997). In contrast, facial micro-expressions refer to brief and involuntary facial changes like the upturn of the inner eyebrows or wrinkling of the nose that happen spontaneously in response to external stimuli, typically over a short time frame of between 65 and 500 ms (Yan et al., 2013). Facial micro-expressions are difficult to fake and can be used to detect genuine emotions (Takalkar et al., 2018). The short duration of these expressions and their subtle movements make it difficult for humans to identify them (Qu et al., 2016); Figure 1 shows some examples of facial micro-expressions compared to facial macro-expressions.

#### 1.1.2. Using Physiological Signals That Cannot Be Faked

This approach relies on physiological responses that are difficult to fake and provide a better understanding of underlying emotions. These responses come from the central (brain and spinal cord) and autonomic nervous systems (regulating body functions like heart rate) (Kreibig, 2010). Electroencephalography (EEG) is one of the methods for measuring brain activity that is commonly used in emotional studies (Alarcao and Fonseca, 2017). Galvanic Skin Response (GSR) and Heart Rate Variability (HRV) can also be used to reliably measure emotional state and have been used widely in emotion recognition studies (Perez-Rosero et al., 2017; Setyohadi et al., 2018; Shu et al., 2018). Although EEG and physiological signals are more reliable and can not be controlled or faked by humans (Wioleta, 2013). These signals can be very weak and easily contaminated by noise (Jiang et al., 2020). So, recognizing emotions using only physiological signals can be pretty challenging.

#### 1.1.3. Using a Combination of Various Input Modalities

In this approach, various modalities are combined to overcome the weaknesses of each individual modality. Combining different physiological signals for emotion recognition (Yazdani et al., 2012; Shu et al., 2018) or fusing only behavioral modalities have been widely explored (Busso et al., 2008; McKeown et al., 2011). Recently some studies tried to improve emotion recognition methods by exploiting both physiological and behavioral techniques (Zheng et al., 2018; Huang et al., 2019; Zhu et al., 2020). Many studies used a combination of facial expressions and EEG signals to achieve this improvement (Koelstra and Patras, 2013; Huang et al., 2017; Zhu et al., 2020). Usually, these researchers work on data that has been collected from subjects while they are watching videos or looking at still images (Koelstra et al., 2011; Soleymani et al., 2011). However, people often do not show many facial expressions in these tasks. Therefore, regular facial expression strategies may not be able to accurately recognize emotions. A limited number of studies used facial micro-expressions instead of facial macro-expressions (Huang et al., 2016), but this area still needs more research and exploration.

Moreover, based on the research of Doma and Pirouz (2020), it is not clear when genuine emotion starts. They hypothesized that participants might still be in their previous emotional state during the first seconds of watching video stimuli. While in the last seconds, they may be more immersed in the video and feel genuine emotion. This is because they better understand the video in the final seconds. They found that the last seconds of EEG data were more informative and showed better emotion prediction results. We believe that the peak time of feeling emotions with the most intensity is affected by many factors such as the stimuli flow, participant personality, or previous experiences.

### 1.2. Goals, Overview, and Contributions

Our hypothesis is that by identifying and analyzing the most emotional part of each stimulus or the time of emerging emotions, we can better understand the body's reaction to emotions and create more robust models for identifying emotions. A primary objective of our research is to improve emotion recognition by combining facial micro-expression strategies with EEG and physiological signals.

In this paper, firstly, each facial video is scanned for micro-expressions that roughly indicate the emergence of emotional stimulation. The micro-expression window is used to approximately determine the time of arising emotions. Then we analyze the EEG and physiological data around the emergence of micro-expressions in each trial in comparison to the analysis of the entire trial. Finally, we compare these two strategies and evaluate our methods based on a subject-independent approach. In the end, we present the results, limitations, and future works. We also use the DEAP dataset as a benchmark to evaluate our method. Additionally, we conduct a user study to collect facial video, EEG, PPG, and GSR data while watching a video task similar to the DEAP dataset but with different sensors.

The main contributions of this research are as follows:

- Fusing facial micro-expressions with EEG and physiological signals to recognize emotions.
- Utilizing facial micro-expressions to identify the emotional stimulation or more informative period of data to improve recognition accuracy.
- Creating a new multimodal dataset for emotion recognition using a low-cost and open-source EEG headset.

## 2. Preliminaries

### 2.1. Emotion Models

Some researchers believe that a few universal emotions exist that apply to all ages and cultures (Maria et al., 2019). A deeper understanding of emotion modeling is necessary to avoid making mistakes in emotion recognition and design a reliable system. Researchers have represented Emotions in two ways. The first perspective is the well-known discrete emotion model introduced by Ekman and Friesen (1971) which categorized emotions into six basic types; happiness, sadness, surprise, anger, disgust, and fear. In contrast, the second perspective considers emotions as a combination of three psychological dimensions: arousal and valence and one of dominance or intensity. Earlier research has demonstrated that two dimensions of arousal and valence are sufficient to explain the underlying emotions, which are primarily driven by neurophysiological factors (Eerola and Vuoskoski, 2011). The most common dimensional model used in the literature is Russel's Circumplex Model (Posner et al., 2005), which only uses valence and arousal for representing emotions, where valence represents a range of negative to positive emotions. In contrast, arousal represents a passive to active emotion.

Based on Russel's Circumplex Model, it is incorrect to categorize emotional states into discrete emotions because the human emotional state is always a mixture of several emotions. So, when people report fear as their emotion, it may be a mixture of excitement, joy, and fear or a combination of negative feelings and fear. So, in positive and negative scary situations, the pattern of the brain and physiological signals are not the same, and categorizing them in a single class leads to incorrect recognition. Additionally, the perception of emotions varies widely based on experience, culture, age, and many other factors, which makes evaluation difficult (Maria et al., 2019). Lichtenstein et al. (2008) showed that the dimensional approach is more accurate for self-assessments. Similarly, Eerola and Vuoskoski (2011) found that the discrete emotion model is less reliable than the dimensional model in rating complex emotional stimuli. They also observed a high correspondence between the discrete and dimensional models.

Facial macro-expressions and facial micro-expressions are usually expressed with discrete emotions, and previous studies used the discrete emotion model to evaluate their strategy. However, most research on neurophysiological emotion recognition and the benchmark dataset that we used, used the Circumplex Model to assess their methods. Since the focus of our study is on revealing underlying emotions and used three neurophysiological cues besides facial micro-expressions, we used the two-dimensional Circumplex Model to evaluate our methodology on the benchmark dataset and our dataset.

### 2.2. Emotion Stimulation Methods

There are different ways of inducing emotions. However, the effect of all emotion induction methods is not the same. Siedlecka and Denson (2019) have classified emotional stimuli into five strategies; (1) watching visual stimuli like images and videos, (2) listening to music, (3) recalling personal emotional memories, (4) accomplishing psychological procedures, and (5) imagining emotional scenes. They showed how different types of stimuli could affect various physiological variables differently. Based on their research, visual stimuli are the most effective induction methods used more frequently in the literature. Quigley et al. (2014) have added Words, body movements, physiological manipulators like caffeine, and Virtual Reality (VR). Roberts et al. (2007) also found that dyadic interactions can be considered as an emotion eliciting method.

### 2.3. Facial Micro Expressions

Facial micro-expressions are brief facial movements in response to emotional stimuli which reveal hidden emotions (Ekman, 2003). Micro-expressions have been used in lie detection, security systems, and clinical and psychological fields to reveal underlying emotions (Yan et al., 2013). Lesser movements and shorter duration times are the main characteristics of facial micro-expressions in comparison to macro-expressions (Liong et al., 2015). Yan et al. (2013) studied the duration of micro-expressions and showed that their duration varies between 65 and 500 ms. Since video episodes are dynamic, long-lasting emotional stimuli, they have been used in micro-expression studies and creating most of the micro-expressions datasets (Li et al., 2013; Yan et al., 2014). To prevent facial macro-expression contamination in micro-expression recording, in many studies, participants are asked to inhibit any facial movements and keep a poker face when watching video (Li et al., 2013; Yan et al., 2013, 2014). However, suppression is brutal to achieve in response to emotional video stimuli (Yan et al., 2013).

A micro-expression has three phases; the onset, apex, and offset phases. In response to emotional stimuli, rapid muscle movements happen in the onset phase, which is involuntary and shows genuine emotional leakage. Sometimes these responses last for a moment as the apex phase. Finally, the emotional reactions disappear in the offset phase, and the face returns to a relaxed state. Returning to a relaxed state may take longer for some people because of natural skin tension or may not happen because of merging with the subsequent emotional stimuli (Yan et al., 2013). The first frame of the onset phase indicates the onset frame in a recorded video, while the frame with the most expressive emotion is the apex frame. The offset frame is when the expression disappears (Goh et al., 2020).

Recognizing emotions using facial micro-expressions has two main steps. The first step is spotting or locating the frame or frames with micro-expressions in a video sequence. The second step is recognizing the micro-expression emotional state (Oh et al., 2018; Tran et al., 2020). Several works have used hand-crafted strategies like Local Binary Pattern with Three Orthogonal Planes (LBP-TOP) (Pfister et al., 2011) or Histogram of Oriented Gradients (HOG) (Davison et al., 2015) to extract features from frames for spotting and recognizing emotions. (Guermazi et al., 2021) proposed an LBP-based micro-expression recognition method to create a low-dimensional high correlated representation of the facial video and used a Random Forest classifier to classify micro-expressions.

Recently, deep learning techniques have been used to extract deep features and classify emotions using facial micro-expressions (Van Quang et al., 2019; Tran et al., 2020). Hashmi et al. (2021) proposed a lossless attention residual network (LARNet) for encoding the spatial and temporal features of the face in specific crucial locations and classifying facial micro-expressions. Although they achieved a promising recognition of emotions in real-time, their model was efficient only when the frame rate was more than 200 fps. Xia et al. (2019) proposed a recurrent convolutional neural network (RCN) to extract spatiotemporal deformation of facial micro-expressions. They used an appearance-based and a geometric-based method to transform facial sequence into a matrix and extract the geometric features of facial movements. They evaluated their strategy based on both leave-one-video-out (LOVO) and leave-one-subject-out (LOSO) approaches and achieved satisfactory results. Similarly, Xia et al. (2020) proposed an RCN network to recognize micro-expressions across multiple datasets. They also discussed the effect of input and model complexity on the performance of deep learning models. They showed that lower-resolution input data and shallower models are beneficial when running models on a combination of datasets.

Ben et al. (2021) reviewed available datasets of facial micro-expressions and discussed different feature extraction methods for recognizing facial micro-expressions. In this research, they introduced a new dataset of micro-expressions and discussed the future directions for micro-expressions research. Similarly, Pan et al. (2021) summarized and compared the available spotting and micro-expression strategies and discussed the limitations and challenges in this area. Detecting facial micro-expressions has received growing attention. Many datasets have been created, and spotting and recognition methods have developed significantly. However, recognizing facial micro-expressions still faces many challenges (Weber et al., 2018; Zhao and Li, 2019; Tran et al., 2020). Oh et al. (2018) discussed various challenges in the dataset, spotting, and recognition areas. They showed that handling facial macro movements, developing more robust spotting strategies, and ignoring irrelevant facial information like head movements and cross-dataset evaluations still needs more attention and research.

### 2.4. Electroencephalography (EEG) Signals

Recently, many neuropsychological studies have investigated the correlations between emotions and brain signals. Electroencephalography (EEG) is one of the neuro-imaging techniques that reads brain electrical activities through electrodes mounted on the scalp. EEG devices differ based on the type and number of electrodes, the position of electrodes (flexible or fixed position), connection type (wireless or wired), type of amplifier and filtering steps, the setup, and wearability (Teplan et al., 2002). EEG devices with higher data quality like g.tec^1^ or Biosemi^2^ or EGI^3^ are usually expensive and bulky and require a time-consuming setup. Alternatively, there are some EEG devices with lower data quality, like the Emotiv Epoc^4^ or MindWave^5^. These EEG devices are affordable and are wireless devices that require less setup time (Alarcao and Fonseca, 2017). OpenBCI^6^ provides a lightweight and open-source (hardware and software) EEG headset, which is positioned in between these two product categories. It captures high-quality data while it is low-cost and easy to set up. Nowadays, because of the improved wearability and lower price of EEG devices, recognizing emotions using EEG signals has attracted many researchers (Alarcao and Fonseca, 2017).

EEG-based emotion recognition is an exciting and rapidly growing research area. However, due to the weak amplitude of EEG signals, it is challenging to recognize emotion using EEG (Islam et al., 2021). Some research has focused on extracting hand-crafted features and using shallow machine learning methods to classify emotions in different application areas like health-care (Aydın et al., 2018; Bazgir et al., 2018; Pandey and Seeja, 2019a; Huang et al., 2021). Several review studies have discussed the effect of various hand-crafted features like brain band powers as well as using various classifiers like Support Vector Machine (SVM) or Random Forest (RF) for recognizing emotions. For instance, Alarcao and Fonseca (2017) reviewed EEG emotion recognition studies. They discussed the most common data cleaning and feature extraction that have been used in the literature for emotion recognition. Based on their review, brain band powers, including alpha, beta, theta, gamma, and delta bands, are effective features for emotion recognition. Similarly, Wagh and Vasanth (2019) provided a detailed survey on various techniques involved in the analysis of human emotions based on brain-computer interface and machine learning algorithms.

Recently many researchers have used raw EEG signals and applied deep learning methods to extract deep features and recognize emotions (Keelawat et al., 2019; Aydın, 2020). Sharma et al. (2020) used an LSTM-based deep learning method to classify emotional states based on EEG signals. Topic and Russo (2021) used deep learning to extract the topographic and holographic representations of EEG signals and classify emotional states. EEG-based emotion recognition methods have been comprehensively reviewed by Islam et al. (2021). They discussed various feature extraction methods and shallow and deep learning methods for recognizing emotions.

Researchers have focused on more advanced network architectures to increase performance in recent years. Li et al. (2021) proposed a neural architecture search (NAS) framework based on reinforcement learning (RL). They trained a Recurrent Neural Network (RNN) controller with an RL to maximize the generated model performance on the validation set. They achieved a high average accuracy of around 98% for arousal and valence on the DEAP dataset in a subject-dependent approach. In another research (Li et al., 2022), they proposed a multi-task learning mechanism to do the learning step for arousal, valence, and dominance simultaneously. They also used a capsule network to find the relationship between channels. Finally, They used the attention mechanism to find the optimal weight of channels for extracting the most important information from data. They reached the average accuracy of 97.25, 97.41% for arousal and valence in the subject-dependent approach. Similarly, Deng et al. (2021) used the attention mechanism to assign weights to channels and then capsule network and LSTM to extract spatial and temporal features. They achieved the average accuracy of 97.17, 97.34% for arousal and valence levels subject-dependently.

### 2.5. Galvanic Skin Responses (GSR) Signals

Previous studies have shown a connection between the nervous system and sweat glands on human skin. Changes in the level of sweat secretion because of emotional arousal lead to changes in skin resistance (Tarnowski et al., 2018; Kołodziej et al., 2019), which is known as the Electrodermal Activity (EDA) or Galvanic Skin Responses (GSR).

When the skin receives the brain's exciting signals caused by emotional arousal, sweating in the human body changes, and GSR signals rise. Kreibig (2010) showed that although EDA signals show changes in emotional arousal, more research is needed to identify the type of emotion using EDA signals. Tarnowski et al. (2018) used GSR local minimum as an indicator for emotional epochs of EEG. They showed that GSR is a good indicator of emotional arousal. In many studies, the GSR signal's statistical features have been used as the features for emotion classification (Udovičić et al., 2017; Yang et al., 2018). Kołodziej et al. (2019) calculated some statistics of peaks (local maxima) and raw GSR signal to use as the feature of signals. They used different classifiers and showed that SVM works better than other classifiers for identifying emotional arousal using these statistical features.

Some studies have used the time series or an averaging signal as the feature vector. Setyohadi et al. (2018) collected the average signal in each second and applied feature scaling. They used this data to classify positive, neutral, and negative emotional states. They used different classifiers, and SVM with Radial Based Kernel showed the best accuracy. Kanjo et al. (2019) used GSR time series and deep learning analysis to understand the valence level during walking in the middle of the city. Ganapathy et al. (2021) showed that Multiscale Convolutional Neural Networks (MSCNN) are effective in extracting deep features of GSR signals and classifying emotions.

In many studies, GSR signals have been used independently for recognizing emotion. But, they are mainly used as a supplementary signal or combined with other physiological signals for recognizing emotion (Das et al., 2016; Udovičić et al., 2017; Wei et al., 2018; Yang et al., 2018; Maia and Furtado, 2019).

### 2.6. Photoplethysmography (PPG) Signals

Photoplethysmography (PPG) is a novel method for measuring Blood Volume Pulse (BVP) using infrared light (Elgendi, 2012). It has been shown that PPG can measure heart rate variability (HRV). HRV is a measure of temporal changes in the heart rate to reveal medical or mental states (Maria et al., 2019). Due to the advent of wearable devices like smartwatches that transmit PPG signals, studies that utilize PPG signals have received more attention. Kreibig (2010) have shown changes in HRV and HR in a different emotional state. Recently a limited number of studies used deep learning strategies to extract deep features of PPG signals. Lee et al. (2019) used a one-dimensional convolutional neural network (1D CNN) to extract deep features of PPG signals and classify emotional states. Similar to GSR signals, PPG data is usually used with other physiological signals to recognize the emotional state.

## 3. Related Works

### 3.1. Multimodal Datasets for Emotion Recognition

Multimodal emotion recognition has attracted the attention of many researchers. A limited number of multimodal datasets with facial video, EEG, and physiological signals for emotion recognition are available for download. The DEAP dataset (Koelstra et al., 2011) and MAHNOB-HCI dataset (Soleymani et al., 2011) are the most popular datasets in multimodal emotion recognition, which include all these modalities. Since EEG signals are sensitive to muscle artifacts (Jiang et al., 2019), these kinds of datasets used passive tasks like watching videos or listening to music to minimize the subject movements.

#### 3.1.1. DEAP Dataset

The DEAP dataset contains EEG data, facial video, GSR, blood volume pressure (BVP), temperature, and respiration data of 32 participants. It used 40 music videos for stimulating emotions, while EEG data were collected using the Biosemi ActiveTwo EEG headset^7^, which has 32 channels. Participants reported their arousal, valence, dominance, and liking level using the self-assessment manikins (SAM) questionnaire (Bradley and Lang, 1994). However, in this dataset, only 22 participants have video data, and for 4 of them, some trials have been missed. The illumination in the facial video is low, and some sensors on the face cover part of the facial expressions.

#### 3.1.2. MAHNOB-HCI Dataset

In the MAHNOB-HCI dataset, eye movements, sound, EEG data, and respiration patterns have been collected for image and video content tagging. After watching video clips, the participants reported their emotional state using the valence-arousal model. Thirty participants were recruited to create this dataset. The Biosemi active II EEG headset^8^ with 32 channels was used for collecting the EEG data.

### 3.2. Exploring the Relationship Between Modalities

Some studies focused on the relationship between behavioral responses and physiological changes in multimodal emotion recognition. For example, Benlamine et al. (2016) and Raheel et al. (2019) used EEG signals to recognize facial micro-expressions. Hassouneh et al. (2020) used single-modality strategies for recognizing emotion in physically disabled people or people with autism using EEG and facial data. Although they did not use multimodal strategies, they showed that emotion could be recognized successfully using each facial expression or EEG signal. They achieved an accuracy of 87.3% for EEG and 99.8% for facial micro-expression from their experimental dataset.

Sun et al. (2020) investigated a strong correlation in emotional valence between spontaneous facial expression and brain activities measured by EEG and near-infrared spectroscopy (fNIRS). However, Soleymani et al. (2015) argued that although EEG signals have some complementary information for facial expression-based emotion recognition, they cannot improve the accuracy of the facial expression system. However, later studies showed improvement by combining EEG and facial expressions. The following section describes these studies.

### 3.3. Fusing Behavioral and Physiological Modalities

In many studies, researchers have shown the impact of emotional stimuli on physiological changes like heart rate, body temperature, skin conductance, respiration pattern, etc. However, they could not identify which emotions had been aroused. Some studies showed that combining physiological emotion recognition and behavioral modalities improves recognition outcomes. Combining facial expressions with physiological modalities attracted the focus of some researchers in this area. Most of these studies focused on traditional facial expression methods and used all recorded video frames to recognize emotions. For example, Koelstra and Patras (2013) used a combination of EEG and facial expressions to generate affective tags for videos. They extracted the power spectral density of power bands and the lateralization for 14 left-right pairs and extracted 230 features of EEG data. They tried to recognize the activation of action units frame-by-frame and finally extracted three features from them for each video. They used feature-level and decision-level fusion strategies. Based on their results, fusion strategies improved tagging performance compared to a single modality. By fusing EEG and face data, arousal accuracy was improved to 70.9% from 64.7% for EEG and 63.8% for the face. This improvement was from 70.9% for EEG and 62.8% for face to 73% by fusion for valence values.

Huang et al. (2017) investigated fusing facial macro-expressions and EEG signals for emotion recognition at the decision level. They used a feed-forward network to classify basic emotions in the extracted face of each video frame. They used their experimental data in this study and achieved 82.8% accuracy in a subject-dependent strategy when fusing EEG and facial expressions. Later, they extended their work by improving facial expression recognition using a CNN model (Huang et al., 2019). They pre-trained a model using the FER2013 dataset (Goodfellow et al., 2013) and used wavelets for extracting power bands and SVM classification for the EEG data. They achieved 80% accuracy for valence and 74% for arousal on the DEAP dataset using a subject-dependent strategy in a multimodal approach. In a similar study, Zhu et al. (2020) used a weighted decision level fusion strategy for combining EEG, peripheral physiological signals, and facial expressions to recognize the arousal-valence state. They used a 3D convolutional neural network (CNN) to extract facial features and classify them, and they also used a 1D CNN to extract EEG features and classify them. They achieved higher accuracy when combining facial expressions with EEG and physiological signals. Chaparro et al. (2018) also presented a feature-level fusion strategy for combining EEG and facial features (using 70 landmark coordinates) to improve recognition results.

In most multimodal emotional datasets' recorded video, no expressions could be observed in many frames. These datasets use passive tasks like video watching to stimulate emotions, so emotional faces can be seen in only a small portion of frames. So, considering all frames in the data analysis or using a majority vote among frames without considering this issue cannot produce a good emotion recognition result. However, many micro-expression can be observed in response to these passive tasks. To the best of our knowledge, only Huang et al. (2016) considered the presence of neutral faces and subtle expressions. They extracted Spatio-temporal features of all frames based on Local Binary Patterns (LBP) strategies. They then trained a linear kernel SVM using these features to calculate expression percentage features and used this feature vector for emotion classification. They extracted all frequencies and frequency bands and then used the ANOVA test to select a subset of these features for EEG. For facial classification, they used the K-Nearest-Neighbor (KNN) classifier for EEG and Support Vector Machine (SVM). They showed that a decision-level fusion strategy works better than a single modality or feature fusion. They achieved an accuracy of 62.1 and 61.8% for valence and arousal, respectively.

Table 1 summarizes the most recent related works. As can be seen, a limited number of studies combined facial expressions with EEG data. Most previous works evaluate their methods subject dependently or cross-subject when there are some trials of all participants in the training and test sets. Although designing general models that identify emotions in unseen participants is extremely useful in our daily lives, only a few studies have used a subject-independent approach to design and evaluate their methods. The accuracy of subject-independent methods is low compared to subject-dependent and cross-subject evaluations and needs more research and exploration. Also, although some research has focused on combining facial expressions with physiological signals, most of them have been designed and trained based on intense facial expressions. While in most used multimodal datasets, people are not allowed to show intense expressions.

**Table 1:** Comparison of recent related works that used the DEAP or MAHNOB-HCI datasets.

**References**	**Modalities**	**Dataset**	**Classification method**	**Evaluation**	**Arousal**	**Valence**
Zhu et al. (2020)	EEG, FE, Physiological	DEAP	DNN	Cross-subject	72.20	78.47
Huang et al. (2019)	EEG, FE	DEAP	EEG SVM FE: CNN	Dependent	74.00	80.00
Pandey and Seeja (2019b)	EEG	DEAP	DNN	Independent	61.25	62.50
Li et al. (2018)	EEG	DEAP	SVM	Independent	–	59.60
Lan et al. (2018)	EEG	DEAP	DA	Independent	–	48.93 for 3 levels
Kwon et al. (2018)	EEG, GSR	DEAP	CNN	Dependent	76.56	80.46
Rayatdoost and Soleymani (2018)	EEG	DEAP MAHNOB	NN	Independent	55.70 61.46	59.22 71.25
Huang et al. (2016)	EEG, FE	MAHNOB	SVM/KNN	Independent	63.22	66.28
Koelstra and Patras (2013)	EEG, FE	MAHNOB	GNB	Dependent	73.00	70.90

*Three evaluation methods have been considered including subject-dependent (dependent), subject-independent (independent), and cross-subject (when there are some trials of all subjects in both the training and test sets). FE, facial expression; SVM, support vector machine; GNB, Gaussian Naive Bayes; KNN, K-nearest neighbor; DNN, deep neural network; CNN, convolutional neural network; DA, domain adaptation. The arousal and valence scores are the percentage accuracy of recognition*.

This research addresses this gap by investigating the best ways to use facial micro-expression strategies combined with EEG and physiological signals for multimodal emotion recognition. We also explore how facial micro-expressions can be used to identify the most emotional part of the facial video, EEG, and physiological data. Furthermore, we create a new dataset of facial video, physiological signals, and EEG signals, which helps develop robust models for emotion recognition. We also explore the performance and quality of collected data from the OpenBCI EEG headset, a low-cost EEG headset for recognizing emotions. Moreover, we propose our strategy of using multimodal data for emotion recognition and finally evaluate it. Overall, the main novelty of this research is fusing facial micro-expressions recognition with the EEG and physiological signals. Another significant contribution of this work is using facial micro-expression to identify a neutral state vs. an emotional state to improve emotion recognition using the EEG and physiological signals.

## 4. Experimental Setup

We created a new multimodal dataset for emotion recognition using lightweight wearable devices and a webcam. We recruited 23 volunteers (12 female and 11 male) aged between 21 and 44 years old (μ = 30, σ = 6) from university students and staff. We only targeted four of the six basic emotions, including happiness, sadness, anger, and fear, plus a neutral state. We collected facial video, EEG, PPG, and GSR signals in a watching video task. We used the arousal-valence model for measuring emotions, and self-report data was also used as the ground truth.

### 4.1. Study Design

**Figure 2:**
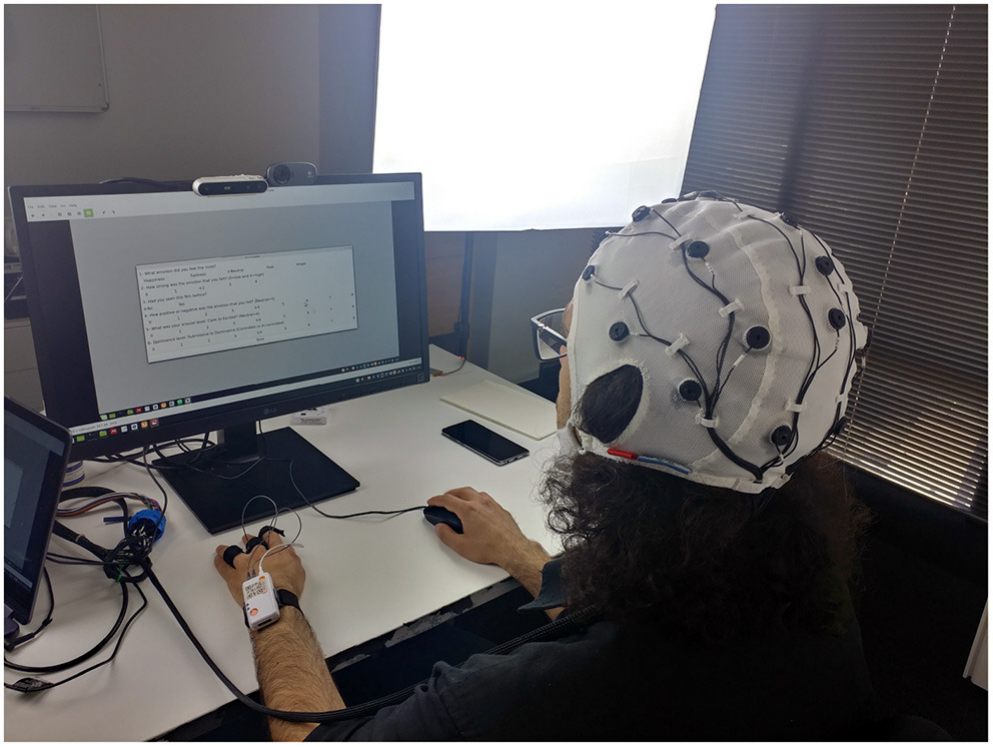
The experiment setup.

The data collection was performed in a room with a controlled temperature. We turned off the room lights, closed the door and curtains, and used two soft-boxes lightings facing the participant to control illumination. One Intel Realsense camera with a frame rate of 30 Hz was used to record facial expressions. Participants wore the OpenBCI EEG soft headset^9^ with the cyton-daisy board to record EEG signals. A Shimmer3 sensor^10^ was used to record PPG and GSR data. Figure 2 shows the experiment setup. Participants wore the Shimmer sensor as a wristband, with PPG and GSR sensors attached to their three middle fingers. We used an Asus laptop (TP410U) to run the experiment scenario and record data. We designed the Octopus-Sensing library, a multi-platform, open-source python library^11^, to create the scenario and simultaneously record data and send synchronization markers to the devices.

### 4.2. Stimuli Set

We considered happiness, sadness, anger, fear, and neutral emotions and used two video clips to stimulate each emotion. Ten video clips with the same length of 80 s were shown in a random order for emotion stimulation. We tried to choose videos with strong emotional scenes and subjects. Most of these videos have been used in previous emotion studies. Table 2 shows the list of movies, their references, and their details.

**Table 2:** The video stimuli set for inducing emotion.

**Emotion**	**Movie**	**Scene**	**References**
Happiness	Pursuit of happiness	Offering job	–
Happiness	Benny and Joone	Benny (Johnny Depp) plays the fool in a coffee shop	Schaefer et al. (2010)
Sadness	The Champ	A kid cries at father's death	Gross and Levenson (1995)
Sadness	E.T.	Saying goodbye	Uhrig et al. (2016)
Fear	Silence of the Lambs	Darkness, chasing	Gross and Levenson (1995); Schaefer et al. (2010)
Fear	Chucky 2	Chucky beats Andy's teacher with a ruler	Schaefer et al. (2010)
Anger	My Bodyguard	Bullying scenes	Gross and Levenson (1995)
Anger	Cry freedom	Police abuse protesters	Gross and Levenson (1995)
Neutral	Weather news	News	Droit-Volet et al. (2011)
Neutral	Documentary	Documentary about soil	–

### 4.3. Scenario

Each session started with introducing the devices, questionnaire, the purpose of the experiment, the meaning of arousal and valence levels, and the overall experiment process for the participant. Then, the EEG headset was placed on the participant's head, a shimmer3 wristband was worn on the participant's non-dominant hand, and the Shimmer's PPG and GSR sensors were attached to their three middle fingers. While watching the videos, participants were asked not to move their heads or bodies and put their hands with the Shimmer3 sensor on a table or on their legs.

**Figure 3:**
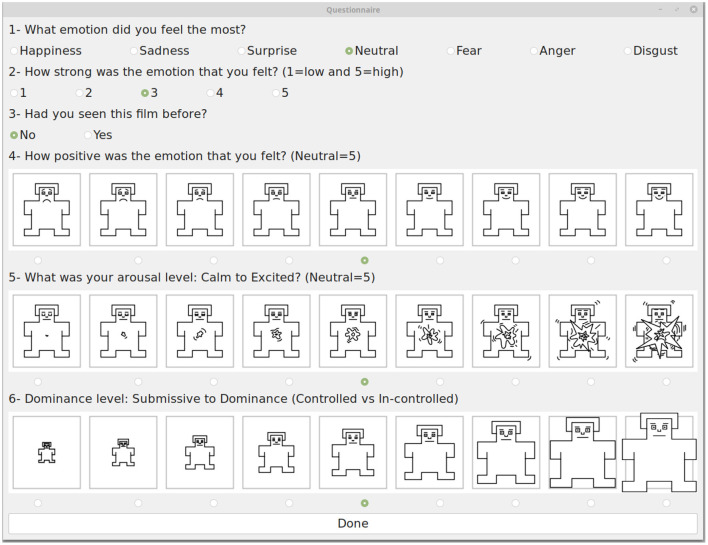
Experimental questionnaire (Three last questions are from SAM questionnaire).

The 10 videos were shown to the participants in random order. The experiment started with showing a gray screen for 5 s, then a fixation cross for 3 s, and then the video was displayed for 80 s. After each video, participants reported their emotional state by filling out a questionnaire similar to the SAM questionnaire (Figure 3) and then moving to the next video by pressing a button. Since the emotional effect of some video clips may have remained for a while, we asked participants to move to the next video clip after resting for a while and when they felt that they were in a neutral state. We left the participant alone in the room during the experiment to prevent any distraction or psychological effects of a stranger's presence.

## 5. Methodology

### 5.1. Ground Truth Labeling

We used self-report data from the SAM questionnaire for ground truth labeling. We only used the reported arousal and valences for the DEAP and our datasets. To classify arousal and valence levels, although there are nine levels for arousal and valence in the SAM questionnaire, similar to previous studies, we used binary classification. We considered five as the threshold for creating binary labels, corresponding to high and low arousal and valence values.

Table 3 shows the average of self-report ratings for arousal and values when rating values were between 1 and 9. This table also shows the percentage of participants who reported each emotion for each video clip. For example, 78.9% of participants reported happiness for the Pursuit of Happiness video clip, and only 4.3% reported fear, 7.8% reported neutral, and 8.7% reported sadness for this video clip. As can be seen, most of the participants reported the target emotion for all stimuli. Although we included all basic emotions in the self-report questionnaire, none of the participants reported other emotions except those in our target emotion list. So, we did not include other emotions in this table and in our evaluation results.

**Table 3:** The Mean arousal and valence rating values and the percentage of participants who reported each emotion for each video-clip in our dataset.

	**Target emotion**	**Valence**	**Arousal**	**Anger**	**Fear**	**Happiness**	**Neutral**	**Sadness**
**Pursuit of happiness**	Happiness	6.83	5.48	0.00	4.30	**78.30**	8.70	8.70
**Benny and Joone**	Happiness	6.57	6.22	0.00	0.00	**87.00**	13.00	0.00
**The champ**	Sadness	3.35	5.17	4.30	0.00	0.00	0.00	**95.70**
**E.T**.	Sadness	5.61	4.57	0.00	4.30	26.10	13.00	**56.50**
**Silence of the lambs**	Fear	4.35	2.82	0.00	**69.60**	0.00	30.40	0.00
**Chucky 2**	Fear	3.83	7.22	0.00	**91.30**	8.70	0.00	0.00
**My bodyguard**	Anger	3.83	5.61	**73.90**	4.30	0.00	8.70	13.00
**Cry freedom**	Anger	3.26	6.35	**60.90**	0.00	0.00	0.00	39.10
**News**	Neutral	4.74	4.35	8.70	0.00	4.30	**78.30**	87.00
**Documentary**	Neutral	5.65	4.52	0.00	0.00	30.40	**69.60**	0.00

*The bolded values prove that for each stimulus, most people reported the target emotion of stimuli*.

### 5.2. Imbalanced Data

In the DEAP dataset, the total number of low and high classes for all participants' trials for valence were 339 and 381, and for arousal, 279 and 444. These values for our dataset for valence classes were 100 and 130, and for arousal were 94 and 136 for low and high classes, respectively. As can be seen, both datasets were not balanced among classes. Also, we used a leave-some-subject-out strategy for splitting the training and test data. Hence, the imbalance state among the training and test sets for each set depended on the participants' rating. We used cost-sensitive learning (Ling and Sheng, 2008) to handle the imbalanced data. Cost-sensitive learning used the costs of prediction errors during the model training. It employed a penalized learning algorithm, which raised the cost of classification errors in the minority class. We used the Scikit-learn library^12^ to measure class weights and used the estimated weights while training the models. We also used the cost-sensitive SVM and RF to handle imbalanced data.

### 5.3. Video Emotion Recognition

**Figure 4:**
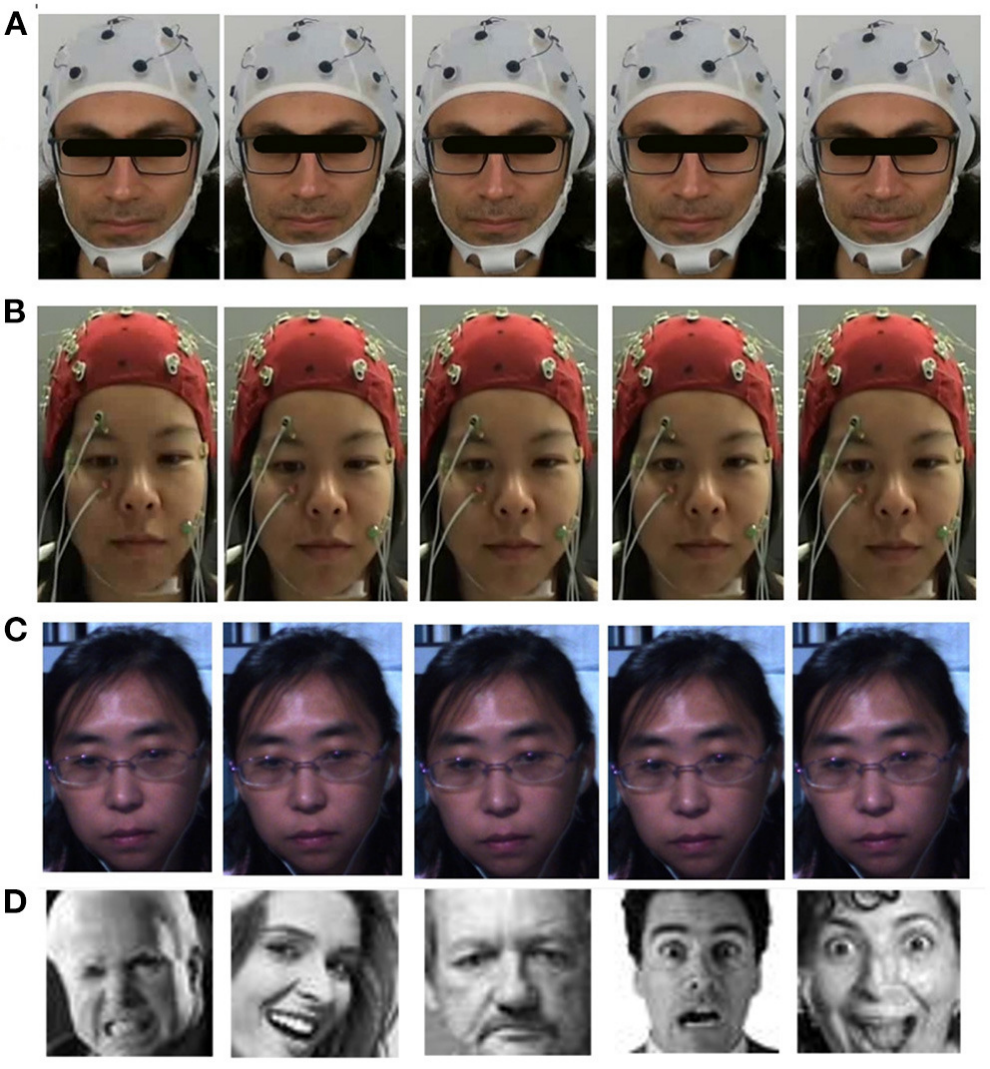
Six frames of a trial of three different datasets from a watching video task. **(A)** Our dataset, micro-expressions in eyes and lips, **(B)** DEAP, micro-expressions around lips, **(C)** SMIC, micro-expressions on the forehead around the eyebrows, **(D)** FER2013, macro-expressions.

In the DEAP dataset and our dataset, we asked participants to keep a poker face while watching videos because of the sensitivity of EEG signals to muscle artifacts. This condition is entirely the same as micro-expression datasets. In micro-expression datasets, participants were asked to inhibit their expressions and keep a poker face while watching the videos to prevent macro-expression contamination (Goh et al., 2020). This condition leads to neutral faces in almost all frames, and only genuine emotions will leak as micro-expressions. Figure 4 shows some frames of a trial from the DEAP dataset, our dataset, the SMIC dataset (Li et al., 2013) and some images from FER2013 dataset (Goodfellow et al., 2013). The SMIC dataset has been specifically collected for facial micro-expression emotion recognition studies. As seen in all of these datasets, emotions can hardly be noticed, and we mostly saw a neutral face. In contrast, in the facial macro-expressions datasets like FER2013 (Goodfellow et al., 2013) and CK+ (Lucey et al., 2010), there are sets of faces with intense expressions (Figure 4).

**Figure 5:**
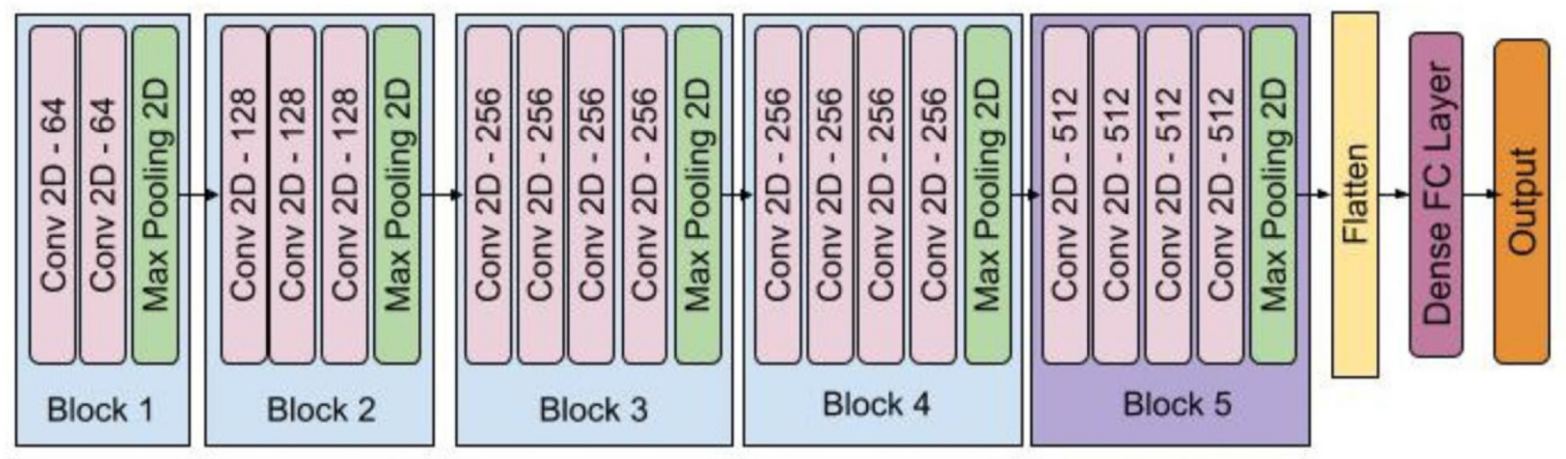
Facial macro-expression model.

We trained a deep convolutional neural network using the FER2013 dataset, tested it on all trials' frames, and mainly got neutral emotions from facial expression recognition. The model had five blocks of convolutional and pooling layers, and its structure was similar to the VGG-16 (Simonyan and Zisserman, 2014) with some extra layers in each block. Figure 5 shows the structure of model. FER2013 is a large-scale dataset automatically collected by the Google image search API and has been widely used in facial emotion recognition studies. It contains 28,709 training images, 3,589 validation images, and 3,589 test images with seven expression labels: anger, disgust, fear, happiness, sadness, surprise, and neutral. We preprocessed the data by converting the images to grayscale images, extracting the face area using the face detection module from the Dlib library, normalizing and resizing them, and finally, feeding them to the deep convolutional network. We removed the non-detected faces from the training and test set and achieved 85% accuracy on the FER2013 test set data. We used the trained model for detecting emotions from each recorded video frame in the DEAP dataset and our dataset. Using the trained model, we applied the same preprocessing steps and predicted each frame's emotion.

Table 4 shows the result of prediction for the DEAP and our datasets. As can be seen, based on the majority vote strategy of all frames' emotions, the detected emotion for 100% of DEAP's trials and 89.1% of the experimental's trials is neutral. For a limited number of participants, the neutral faces were mistakenly predicted as sadness emotion in all trials.

**Table 4:** The result of using facial macro-expression model for detecting emotions from all frames.

	**Percentage of trials with all neutral frames**	**Percentage of trials with majority vote neutral**	**Percentage of frames with detected emotion**						
			**Anger**	**Disgust**	**Fear**	**Happiness**	**Neutral**	**Sadness**	**Surprise**
**DEAP dataset**	80.3	100	0.0	0.0	0.0	0.0	98.7	1.3	0.0
**Our dataset**	6.9	89.1	0.0	0.0	0.0	0.0	87.5	12.5	0.0

This result shows that neutral faces or faces with subtle or micro-expressions cannot be easily identified with facial macro-expressions methods. Since the condition of recorded video in the DEAP and our datasets is the same as the micro-expression datasets, we used micro-expression methods to detect facial video expressions in these two datasets and investigated their performance. So we considered the facial data in the DEAP and our dataset as facial data with micro-expressions and used a facial micro-expression strategy for video-emotion recognition.

We used a two-steps facial micro-expression recognition strategy. Firstly, we used an automatic spotting strategy to automatically find the apex frame based on maximum facial components' movements compared to the first and last frame of the trial. Then we extracted a set of frames around the apex frame and considered these frames instead of the overall video for classification. Finally, we fed the extracted sequence to a 3D convolutional neural network.

**Figure 6:**
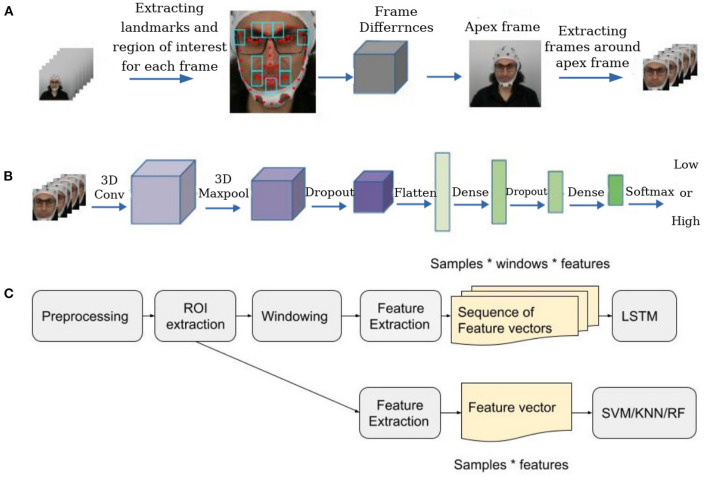
Emotion recognition strategy. **(A)** Locating the apex frame in the sequence of all frames in each trial. **(B)** The architecture of the network for detecting arousal or valence states in facial videos based on micro-expressions. **(C)** The overall structure of EEG and physiological classification.

To prepare frames for spotting micro-expressions, first of all, we employed a pre-trained YOLO v3 network (Redmon and Farhadi, 2018) on the WIDER FACE dataset (Yang et al., 2016) for face detection. We chose the WIDER FACE dataset because it contains images with varying degrees of scale, occlusion, and poses, enhancing the feature space for the model to learn better and giving better real-time performance under any condition. Then we followed the spotting method introduced in Van Quang et al. (2019) to identify the apex frame (frame with micro-expression) in each video. In this spotting method, firstly, we extracted ten regions of the face around facial components where muscle movements occur very frequently. For the next step, we considered the first frame of the video sequence as the onset frame and the last frame as the offset frame and calculated the absolute pixel differences between each frame and the onset and offset frames in the ten regions. Finally, we calculated the per-pixel average value for each frame. We considered the frame with the higher intensity differences as the apex frame. We considered a window of frames around the apex frame as the region of interest (ROI) and only used these frames in the classification step (Figure 6A).

Although the recorded videos in our dataset and the DEAP dataset are longer and may contain more neutral frames than facial micro-expressions datasets, facial micro-expression spotting methods could still find the apex frame. So we still have the onset, apex, and offset frames. There may be several neutral frames before the actual onset frame and after the offset frame, but all of them are the same and will not affect the result of the spotting algorithm. This is because the actual onset frame and the first frame or the real offset frame and the last frame are almost the same. Hence the measured absolute pixel differences between the apex frame and actual onset or offset frame will be practically the same as those between the apex frame and the first or last frame. Although there may be more facial expressions and apex frames in the video, the actual offset frame, and the last frame are nearly identical since both frames depict the face in a neutral state.

We considered different window sizes for the ROI and discussed it in the result section. Figure 4 illustrates six frames of extracted sequences around the apex frame for DEAP and our dataset, in addition to a sequence of the SMIC dataset. We used a 3D Convolutional Neural Network (3D CNN) to classify micro-expression sequences. It is one of the state-of-the-art models in micro-expression emotion recognition (Reddy et al., 2019) which achieved good performance on two popular micro-expressions dataset CASME II (Yan et al., 2014), and SMIC (Li et al., 2013). This method achieved 87.8% accuracy on the CASME II dataset and 68.75% accuracy on the SMIC dataset. We used this model to extract deep features and classify micro-expressions in the DEAP and our datasets. Since the ground-truth labeling in both datasets is based on arousal and valence levels, instead of classifying micro-expressions based on basic emotions, we classified micro-expressions based on arousal and valence levels. To classify emotional states based on arousal or valence, we applied the model two times to the data, once for classifying arousal levels and once for classifying valence levels. In this model, instead of using six as the output shape in the last dense layer, we used 2 to classify micro-expressions based on low and high arousal or valence.

At first, we used the YOLO face detection algorithm to detect the face in each frame in the ROI, then converted it to the grayscale image, normalized it, and resized it. Finally, we fed the preprocessed sequences into two 3D CNN models introduced in Reddy et al. (2019) for classifying arousal and valence separately. Figure 6B) illustrates the structure of the 3D CNN model.

### 5.4. EEG and Physiological Emotion Recognition

We considered micro-expressions as an indicator for identifying the most emotional time of each trial. Then, we used an ROI-based strategy for recognizing arousal and valence using EEG and physiological data. We considered the time of the apex frame as the most emotional time of each trial. Then, we located corresponding samples in the EEG and physiological data at this time. Due to the difference in sampling rates between EEG, physiological data, and video frames, we multiplied the sampling rate of each signal at this time to determine the ROI. Finally, we extracted a couple of seconds of data around it, considered the extracted part as the ROI, and analyzed only the extracted data. We regarded different window sizes for extracting ROI and discussed it in the result section.

To analyze EEG and physiological data, we followed the main steps of emotion recognition: preprocessing, feature extraction, and classification. Firstly, we cleaned data and then extracted ROI sections and only used ROI data as the input of the feature extraction step. To classify data, we used two methods for classifying EEG and physiological data. In the first method, we extracted some features—described in the following sections—from the whole data or ROI section. We used these features as the input of Support Vector Machine (SVM), K-Nearest Neighbor (KNN) (Bressan and Vitria, 2003) and Random Forest (RF) (Criminisi et al., 2011) classifiers. In the second method, firstly, we partitioned each trial into non-overlapping windows. Then extracted features the same as the previous method from each window and made a sequence of consequences feature vectors. We used these sequences as the input of a stacked Long-Short-Term-Memory (LSTM) network (Staudemeyer and Morris, 2019) with two layers of LSTM to extract temporal features. Finally, we used a Dense layer with Adam optimizer (Kingma and Ba, 2014) to separately classify the data for arousal and valence labels. Figure 6C shows the overall structure of EEG and physiological data analysis.

### 5.5. Data Cleaning

#### 5.5.1. EEG

**Figure 7:**
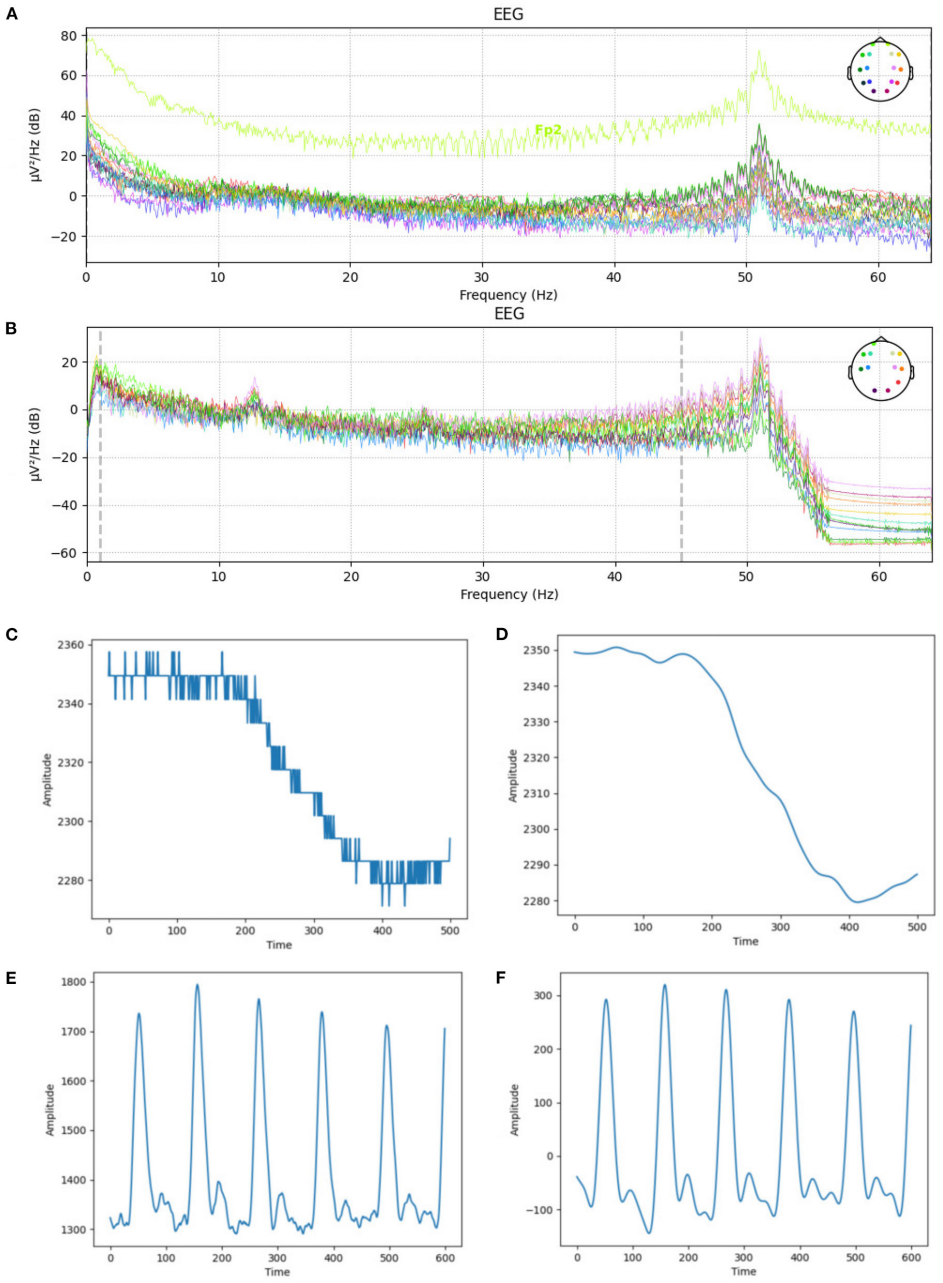
The EEG, GSR, and PPG signals before and after applying preprocessing steps and noise removal. **(A)** EEG channels' frequencies before preprocessing. **(B)** EEG channels' frequencies after preprocessing. **(C)** GSR signal's amplitude before preprocessing. **(D)** GSR signal's amplitude after preprocessing. **(E)** PPG signal's amplitude before preprocessing. **(F)** PPG signal's amplitude after preprocessing.

We used the preprocessed EEG data in the DEAP dataset, removed the first 8 s of data, including 3 s of baseline, and considered 5 s as the engagement time and finally normalized data. The engagement time was chosen by observation and was the average time participants were immersed in the video. For our dataset, we applied bandpass filters and extracted frequencies between 1 and 45 Hz which are the frequency range of brain waves (Huang et al., 2016). Then a common average reference was applied, and finally, we normalized the data. Figures 7A,B show the frequencies of EEG channels before and after data cleaning.

#### 5.5.2. PPG and GSR

A bandpass filter with a low-cut frequency of 0.7 Hz and a high-cut frequency of 2.5 Hz has been used to remove noise from the PPG signals. Similarly, a low-cut frequency of 0.1 and a high-cut frequency of 15 Hz were used to clean the GSR signals. We also used a median filter to remove rapid transient artifacts from the GSR signal. Finally, we normalized these GSR and PPG signals. Figures 7C–F shows the amplitude of one sample of GSR and PPG signals before and after data cleaning.

### 5.6. Feature Extraction

#### 5.6.1. EEG

To extract EEG features, we applied a Fast Fourier Transform (FFT) on each window of data to extract EEG band powers. We made a feature vector of five features by extracting EEG power bands from each window and considered the average of each as one feature. We extracted Delta (1–4 HZ), Theta (4–8 HZ), Alpha (8–12 HZ), Beta (12–30 HZ), and Gamma (30–45) bands. These features have commonly been used in previous studies (Wagh and Vasanth, 2019).

#### 5.6.2. PPG and GSR

We calculated some statistical features for both GSR and PPG signals. The average and standard deviation of the GSR signal and the first and second-order discrete differences of the GSR signal made up the GSR feature vector. To build the PPG feature vector, we considered the average and standard deviation of the PPG signal. The PPG and GSR feature vectors have similar characteristics, so we concatenated the two feature vectors and referred to them as physiological data.

### 5.7. Fusion Strategy

There are several methods for fusing data from various sources. Fusing data can be done mainly in two major ways, (1) feature-level or early fusion and (2) decision-level fusion or late fusion (Shu et al., 2018). We fused the PPG and GSR signals at the feature level, addressed the created features as physiological features, and classified them. We used two different strategies for fusing facial micro-expressions, EEG and physiological classification results in the decision level. The first strategy was based on majority voting, where we selected the prediction that had the most votes among EEG, facial and physiological predictions as the final prediction. In the second strategy, we used the weighted sum of all probabilities as the decision level fusion strategy (Koelstra and Patras, 2013; Huang et al., 2017). We gave various weights in the range [0, 1] with 0.01 steps to these three classifiers, measured the best weights on the training data, and used these weights in the fusion step. The Equation (1), $p_{Modality}^{x}$ shows the probability of each class using a specific modality, and a, b, and c are weights.


(1) $$\begin{array}{r} p_{o}^{x}=a\times p_{Video}^{x}+b\times p_{EEG}^{x}+c\times p_{Physiological}^{x} \end{array}$$

x∈[0,1]

a+b+c=1

## 6. Result and Discussion

### 6.1. Evaluation Strategy

We used a subject-independent strategy to evaluate our methods and find a general model. We used the leave-some-subject-out strategy cross-validation. Since our models were not complex and the size of datasets was not significant, we did not use a GPU for training models. All models were trained on a computer with Gnu-Linux Ubuntu 18.04, Intel(R) Core(TM) i7-8700K CPU (3.70 GHz) with six cores. We randomly shuffled participants into six-folds and trained models for all folds in parallel. For the DEAP dataset, 3 participants were considered in the test set in each fold. In our dataset, four participants were considered in the test set. The reported result is the average of all folds results.

The four main metrics in evaluating models are accuracy, precision, recall, and F-Score or F1. They are measured using the Equation (2) for binary classification. In this section, all of the results are based on F-Score. In these equations, TP is True Positive which means the number of correctly positive class predictions. The True Negative (TN) measures how many correctly negative predictions were made. False Positives are the number of incorrectly predicted positive classes. FN stands for False Negative, the number of incorrectly negative class predictions. We used binary classification for classifying arousal and valence separately and chose the F-Score for evaluating our methods which are appropriate for imbalanced data. (Sun et al., 2009).


(2) $$\begin{array}{r} Accuracy=\left(TP+TN\right)/\left(TP+FP+FN+TN\right) \end{array}$$

Precision=TP/(TP+FP)

Recall=TP/(TP+FN)

F1=2×(Recall×Precision)/(Recall+Precision)

### 6.2. Hyper-Parameter Tuning

To measure the best hyper-parameters for SVM, RF, and KNN, we used grid-search cross-validation parameter tuning (Claesen and De Moor, 2015). Hyper-parameters were tuned using six-fold cross-validation when split data based on the leave-some-subject-out strategy. We got the best result when considering Radial Basis Function (RBF) kernel with 200 as the regulation parameter for SVM, five neighbors for KNN, and 500 estimators for RF. For the 3D convolutional model for micro facial expression, we used the same parameters as the source study (Reddy et al., 2019). We only set the number of epochs to 50. We also empirically found that using two stacked LSTM generates better results when the first LSTM has 80 neurons and the second has 30 neurons. We considered 128, 32, and 64 as the batch size in LSTM model training for EEG, GSR, and PPG classifiers and set the number of epochs to 100 for them. We did not tune the learning rates. Instead, we used a reduced learning rate in the range of 0.001–0.0001, which decreases with a rate of 0.5 when validation loss is not changing.

### 6.3. Identifying ROI Size

Micro-expression duration varies between 65 and 500 ms. This time may increase when the emotion lasts for a while or may merge with the next micro-expression that is the response of the subsequent emotional stimulus (Yan et al., 2013). The DEAP dataset recorded facial data at 50 frames per second. This means that if we consider the length of a micro-expression as half a second, a micro-expression appears in 25 frames when the frame rate is 50 Hz. In our dataset, the frame rate is 30 frames per second, so the length of a micro-expression is 15 frames. We considered two different window sizes, including 20 and 60 frames, around the apex frames to cover short micro-expressions or long-lasting micro-expressions. We considered a bigger window size to cover micro-expressions that remain longer or overlap with the next micro-expression. Table 5 compares the effect of these two window sizes on the prediction result when we want to classify emotions according to arousal and valence levels. As can be seen, the result of 60 frames is better for both datasets. Since increasing the window size increases the probability of including other head movements, adding non-informative data to the sequence, and increasing the computation cost, we did not consider a bigger window size. Table 5 shows the f-score of 3D CNN models from the DEAP and our datasets for these two different window sizes. We used the prediction result of facial micro-expression classification combined with the other modalities at the decision level to classify arousal and valence levels.

**Table 5:** The F-Score of facial micro-expression recognition when the window size is 20 frames around the apex frame or 60 frames.

	**Arousal**		**Valence**	
**Window size**	20	60	20	60
**DEAP**	55.0	**59.0**	55.7	**56.8**
**Experimental**	61.0	**62.2**	57.0	**61.1**

*The bolded values show the highest F-scores for each dataset and emotional level according to the window size. Increasing the window size to 60 frames increase the F-score*.

**Figure 8:**
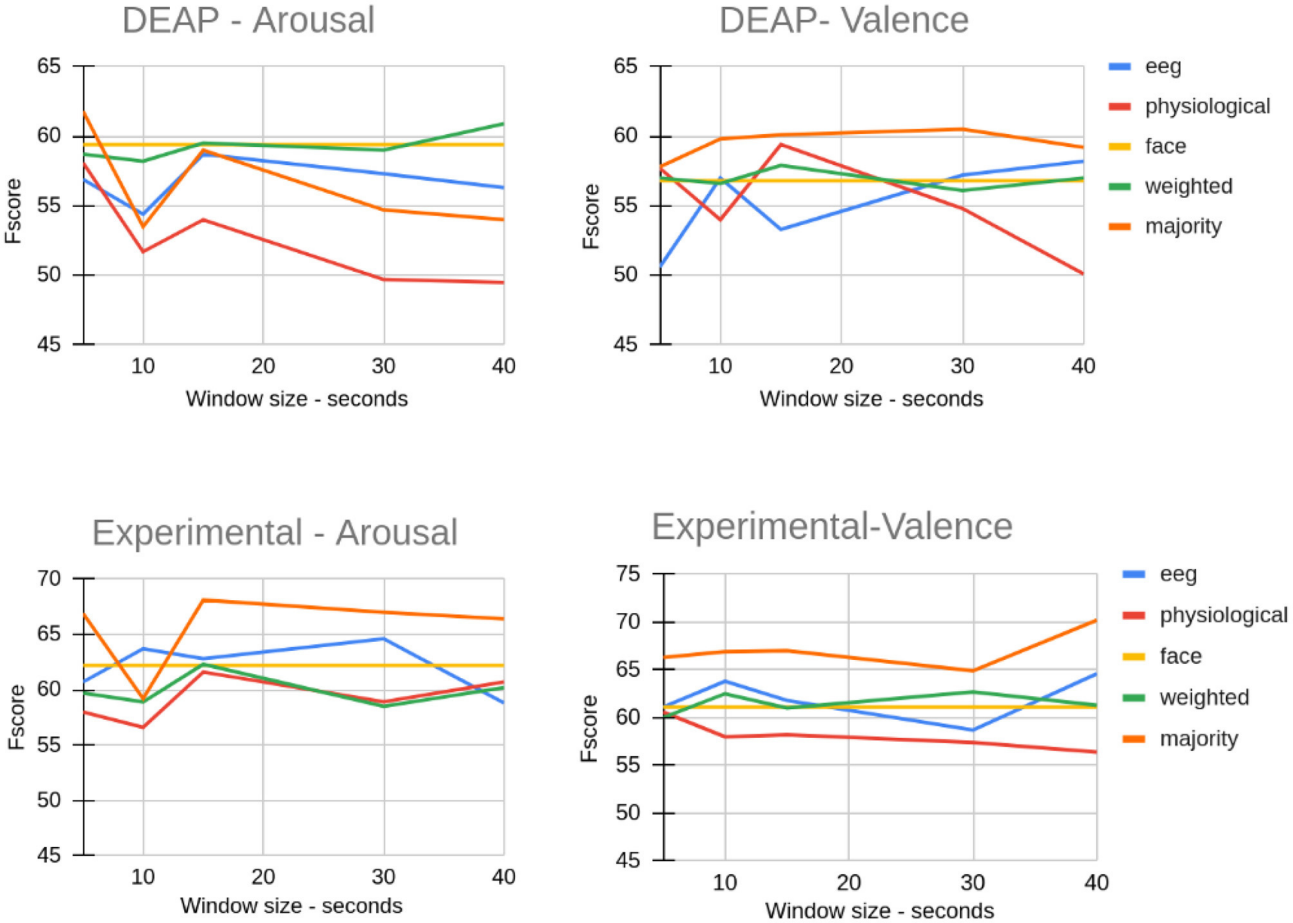
The effect of ROI size on EEG, physiological, and fusion classification on the DEAP dataset and our experimental dataset (Window size 40 means all frames).

We considered various sizes for extracting the ROI from EEG and physiological data and compared the effect of ROI size on the classification result. Figure 8 shows the impact of various ROI sizes on the classification result when we used the LSTM method. The reported values are the average of F-Score values for all folds. As shown in Figure 8, for both datasets, the window size of 15 created almost the highest F-Score when using majority fusion. For the DEAP dataset, weighted fusion created the best result for predicting arousal when we considered all of the data. Despite not seeing any consistent pattern in the two different datasets shown here, assuming a small portion of data in the most emotional part can yield a similar or better result than using all the data. This indicates that if we accurately identify the most emotional part of data, we can accurately study brain and body responses to emotional stimuli.

We also used SVM, KNN, and RF classifiers to classify the ROI section when the window size is 15 and when the whole data was considered. We compared the F-Score of these classifiers with the LSTM method when all data or only the ROI section has been considered for classification in Table 6. The F-Score of facial micro-expression with window size 60 reported in Table 5 has been considered in the fusion strategies. We fused the prediction result of the facial micro-expression method with all classifiers that we used. As can be seen in these tables, the LSTM method achieved the best result in both datasets for arousal and valence. This shows that exploiting both temporal and spatial features could help detect emotions. Also, the result of fusion strategies is considerably better than single modalities. The majority vote fusion in our dataset for arousal and valence, and only valence in DEAP outperforms weighted fusion. Combining PPG and GSR only improves the performance of the LSTM method in classifying valence levels when applied to ROI data. Also, the F-Score of the ROI-based LSTM is relatively close to or sometimes better than using LSTM on the whole of the data. This shows that using a small portion of data can be informative as using all of the data.

**Table 6:**
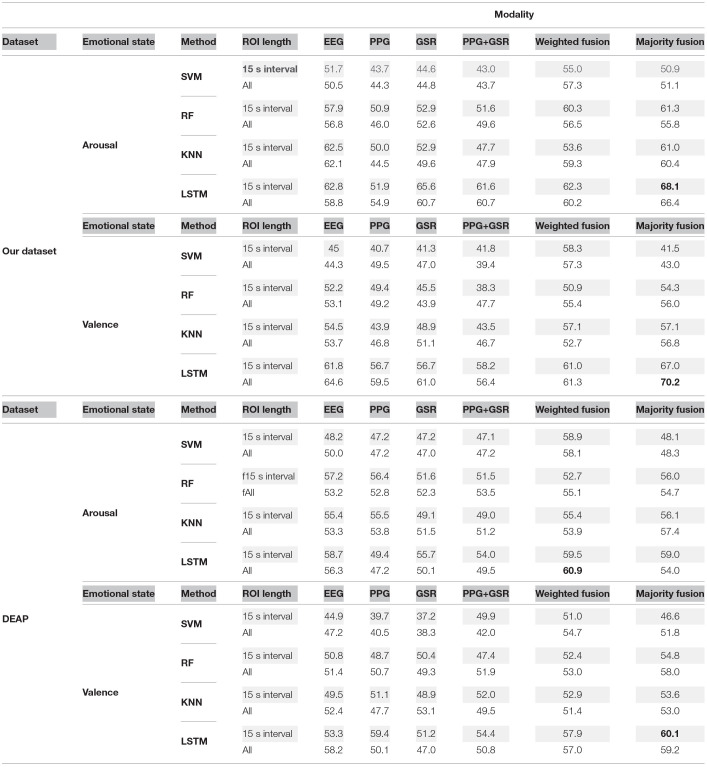
Comparison of F-Score value of LSTM, RF, SVM, and KNN methods when ROI of 15 s or all of data was used.

*The bolded values show the highest F-scores for each dataset and emotional level. The LSTM method shows the best result*.

Although other classifiers generated a good result for some modalities for arousal or valence in one of these datasets, their predictions did not improve after fusing with other modalities compared to the LSTM method. When the prediction accuracy is below or around random prediction or 50% for binary classification, it could not find any particular pattern in the data. Thus, the mismatching between single modalities prediction increases, leading to a degrading f-score in the fusion strategy. According to Table 6, the f-scores of SVM, KNN, or RFC for some modalities were below or around 50%. Therefore, this leads to ineffective fusion.

### 6.4. Computation Cost

Instead of using all frames as the input of the 3D convolutional model, only 60 frames of each video were employed as the model's input. The DEAP dataset has 3,000 frames in each video, and our dataset has 2,400 frames in each video. By extracting micro-expression ROI, we decrease the input size for DEAP with the rate (60/3,000) and our dataset with (60/2,400). This drop-off in input size leads to a considerable decline in computation cost. Our dataset has 230 (23 * 10) trials for all participants, while the DEAP dataset has 720 (18 * 40) trials for all participants. The face model's input for both datasets is (60 * 64 * 64), where 60 is the number of frames in each trial and 64 * 64 is the dimension of the frame in grayscale in the face area. Training six-folds of face models for the DEAP dataset in parallel took 1 h and 37 min (235 s for each epoch). This time was 33 min for our dataset (79 s for each epoch) because of each participant's lower number of trials.

Moreover, despite previous studies which used the LSTM network for classifying EEG signals and feeding raw signals as the input of network (Ma et al., 2019), we extracted a limited number of features from each second of data to decrease the input size. We created a new sequence of data that is considerably smaller than raw data while still being informative. For example, for the EEG data, the size of each trial was (duration in seconds * sampling rate * channels). We decreased this size to (duration in seconds * five power bands). This decrease is the same for physiological data. Training the LSTM models for EEG, PPG, and GSR were done in parallel for six-folds. It took 12 min and 14 s to train all these models for the DEAP dataset, while each epoch took around 1–3 s to run. The training time for our dataset took 5 min, with each epoch taking between 25 and 100 ms to run.

### 6.5. Final Result

Table 7 shows the final results of classifying the ROI section for a single modality or fusion strategy when the ROI window size is 15 s. As can be seen, fusing micro expressions with EEG and physiological signals leads to higher accuracy and F-Score than using a single modality in both datasets. We achieved similar or better accuracy in recognizing arousal and valence levels than related works that used subject-independent strategies.

**Table 7:**
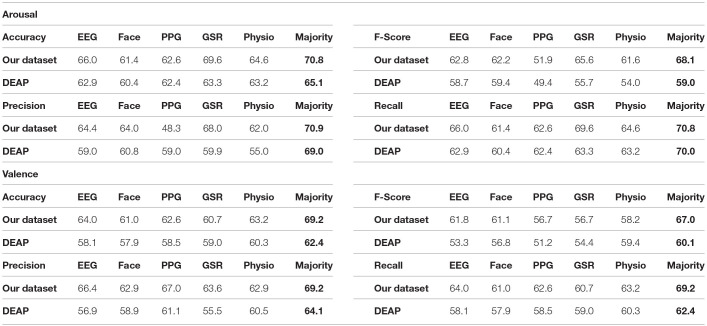
Accuracy, F-score, precision, and recall for arousal and valence.

*The best values for each measurement are bolded in each row for our dataset and DEAP dataset*.

There is not any standard benchmark for evaluating various emotion recognition studies. There are multiple datasets with different scenarios in data collection that record emotional data using various modalities and sensors. The variety in the datasets, emotion models, the way of splitting data, evaluation strategies, and evaluation metrics affect the final emotion recognition results. For this reason, we should consider all these factors for comparing various studies. Compared to the previous work reported in Table 1, the accuracy of the proposed methods is considerably high while considering the subject-independent approach, which is the most challenging evaluation condition.

Although detecting facial micro-expressions is still a big challenge in the literature and needs more exploration, we have shown that it could considerably decrease computational costs for video emotion recognition. There are some challenges for detecting micro-expressions that affect emotion recognition performance, including contamination with other facial movements, pose changes, poor illumination, and the possibility of faked or posed micro-expressions (Zhao and Li, 2019). With the DEAP dataset and our dataset, the chance of faked micro-expressions is low due to the poker face condition. There are, however, some unwanted movements that can affect the results of detecting micro-expressions and identifying regions of interest.

We also found that the low-cost OpenBCI EEG cap could achieve similar performance to the Biosemi Active II cap used in the DEAP dataset. Our result shows that although this tool is low-cost, it can be used as a reliable tool for collecting brain signals for emotion recognition.

Similar to previous studies, our result shows that combining various modalities leads to a better recognition result with a 3–8% improvement after fusion. We achieved 65.1% accuracy for arousal and 69.2% accuracy for valence in the DEAP dataset, which is better than single modalities. These corresponding values are 70.8 and 69.2% for arousal and valence in our dataset. Table 7 shows these improvements. Although there are some disadvantages to employing multimodal data, such as increased computing cost and data analysis complexity, the benefits of enhancing prediction performance outweigh them. Nowadays, most processing systems have multiple cores, making parallel processing easy. We can perform multimodal data analysis using parallel processing at almost the same time as a single modality analysis.

## 7. Limitations

Despite showing that facial micro-expressions can effectively identify emotions, we face some challenges that should be addressed in the future. Due to involuntary facial movements, such as eye blinking, head movements, or regular facial expressions, micro-expressions can be mistakenly detected (Tran et al., 2020). These movements result in incorrect detection of the apex frame. In the future, we could significantly improve the spotting strategy result by introducing new facial micro-expression datasets and using deep learning methods. In this paper, we used a simple traditional micro-expression spotting strategy to detect the apex frame. We showed that facial micro-expressions could be combined with other modalities in emotion recognition. In the future, we want to use more robust spotting strategies to improve recognition quality.

Furthermore, facial micro-expression methods face challenges similar to facial macro-expressions, including illumination conditions, cultural diversity, gender, and age. These limitations can be overcome by using new datasets and more robust deep learning methods. Also, combining facial micro-expressions with physiological signals will improve the recognition result. EEG headsets and physiological sensors are not as accessible as cameras for most people. We are now closer than ever to developing robust models for emotion recognition because more and more affordable and wearable devices like smartwatches, activity trackers, and VR headsets are equipped with physiological sensors. We can achieve this objective by introducing more accurate, wearable, and affordable EEG sensors and developing a more robust algorithm for physiological emotion recognition.

We used a spotting strategy to detect apex frames in our study. Since spotting methods still need more exploration and are an open challenge (Oh et al., 2018), we could improve our results in the future by manually annotating the DEAP and our dataset. Manually annotating these datasets is a labor-intensive and time-consuming activity. Still, because they were collected under similar conditions as micro-expression datasets, we can use them as micro-expression datasets for making more robust micro-expression models.

## 8. Conclusions

It can be essential to accurately recognize emotions for human-human and human-machine applications. The previous techniques relied heavily on facial macro-expressions. This paper demonstrated our strategy for how facial micro-expressions can be used effectively with EEG and physiological signals to recognize emotional states.

In this paper, we used facial micro-expressions emotion recognition instead of facial macro-expressions emotion recognition combined with physiological modalities, which is more reliable in identifying genuine emotions. Also, we used a facial micro-expressions spotting strategy to roughly determine the most emotional and informative part of the data. We identified each trial's region of interest (ROI) using a landmark-based spotting strategy for detecting micro-expressions. Several frames around the micro-expression were extracted and fed to a 3D convolutional network. In addition, we extracted a sequence of feature vectors from EEG and physiological data in the ROI when the data was partitioned into 1 s windows. To extract temporal features from physiological signals and EEG signals, we employed LSTM. We evaluated ROI classification with LSTM, SVM, KNN, and RF classifiers compared to classifying all data. Our methods were evaluated based on a subject-independent approach. According to our results, we could obtain a similar or even better accuracy by using a small portion of data compared to all the data. According to our findings, facial micro-expressions could identify the more emotional part of data with sufficient information and low noise.

Moreover, we used a low-cost, open-source EEG headset to collect multimodal emotional data. We evaluated our method based on the DEAP dataset and our own data. Lastly, we combined multiple modalities and found that fusing their outputs improved emotion recognition. In addition, we found that facial micro-expressions were more effective at detecting genuine emotions than facial macro-expressions methods.

Due to the high data quality and ease of use of the OpenBCI hardware, we want to follow up our study with more data collection with various settings with OpenBCI. The collected data will be used to pre-train the upcoming models to create a robust model for recognizing emotions in EEG data. In the future, after getting ethics approval for publishing the dataset, we want to make the EEG and physiological data publicly available. This will help researchers to train more robust models for emotion recognition.

We would like to examine more features in the future and see if changing the feature set or using more complex features will improve the LSTM method performance. We would also like to use more complex fusion strategies to exploit multimodal sensors effectively.

In addition, another future direction is to explore how facial micro-expressions can be extracted from more natural head movements (for example, not requiring people to maintain a poker face). Moreover, it would be interesting to identify facial micro-expressions in the presence of regular facial expressions and explore how a combination of both could be used to recognize emotions.

Finally, we are interested in incorporating this emotion recognition approach into applications such as healthcare with remote therapy sessions, identifying emotional disorders in patients, or creating intelligent assistants to help patients or the elderly. In addition, this emotion model can be used in our daily interactions with humans, such as enhancing teleconferencing and making remote interactions more immersive. Furthermore, it will improve our interactions with virtual agents and other interactive devices we regularly use by giving them the ability to recognize and respond to our emotions.

## Data Availability Statement

The datasets presented in this article are not publicly available due to privacy concerns. Requests to access the datasets should be directed to zsaf419@aucklanduni.ac.nz.

## Ethics Statement

The studies involving human participants were reviewed and approved by the University of Auckland Human Participants Ethics Committee. The patients/participants provided their written informed consent to participate in this study. Written informed consent was obtained from the individual(s) for the publication of any potentially identifiable images or data included in this article.

## Author Contributions

NS: the main author, conducting the research, data collection, data analysis, and writing. SW: facial micro-expression analysis. KD: facial detection model. AN: technical and writing feedback. SN: co-supervisor and technical feedback. EB: co-supervisor, psychological support, and feedback. MB: main supervisor and lab coordinator, technical, and writing feedback. All authors contributed to the article and approved the submitted version.

## Conflict of Interest

The authors declare that the research was conducted in the absence of any commercial or financial relationships that could be construed as a potential conflict of interest.

## Publisher's Note

All claims expressed in this article are solely those of the authors and do not necessarily represent those of their affiliated organizations, or those of the publisher, the editors and the reviewers. Any product that may be evaluated in this article, or claim that may be made by its manufacturer, is not guaranteed or endorsed by the publisher.

## Footnotes

^1^ https://www.gtec.at/

^2^ https://www.biosemi.com

^3^ https://www.egi.com/

^4^ https://www.emotiv.com/epoc/

^5^ https://store.neurosky.com/pages/mindwave

^6^ https://openbci.com

^7^ https://www.biosemi.com/

^8^ https://www.biosemi.com/

^9^ https://openbci.com/

^10^ http://www.shimmersensing.com/

^11^ https://octopus-sensing.nastaran-saffar.me

^12^ https://scikit-learn.org/stable/index.html
